# A novel laser-collider used to produce monoenergetic 13.3 MeV ^7^Li (d, n) neutrons

**DOI:** 10.1038/srep27363

**Published:** 2016-06-02

**Authors:** J. R. Zhao, X. P. Zhang, D. W. Yuan, Y. T. Li, D. Z. Li, Y. J. Rhee, Z. Zhang, F. Li, B. J. Zhu, Yan F. Li, B. Han, C. Liu, Y. Ma, Yi F. Li, M. Z. Tao, M. H. Li, X. Guo, X. G. Huang, S. Z. Fu, J. Q. Zhu, G. Zhao, L. M. Chen, C. B. Fu, J. Zhang

**Affiliations:** 1 Laboratory of Optical Physics, Institute of Physics, CAS, Beijing 100190, China; 2 Key Laboratory for Laser Plasmas (MOE) and Department of Physics and Astronomy, Shanghai Jiao Tong University, Shanghai 200240, China; 3 Key Laboratory of Optical Astronomy, National Astronomical Observatories, CAS, Beijing 100012, China; 4 Institute of High Energy Physics, CAS, Beijing 100049, China; 5 CoReLS, Institute for Basic Science, Gwangju 61005, Korea; 6 NDC, Korea Atomic Energy Research Institute, 34057, Korea; 7 Department of Astronomy, Beijing Normal University, Beijing 100875, China; 8 Shanghai Institute of Laser Plasma, Shanghai 201800, China; 9 National Laboratory on High Power Lasers and Physics, Shanghai 201800, China; 10 Collaborative Innovation Center of IFSA, Shanghai Jiaotong University, Shanghai 200240, China

## Abstract

Neutron energy is directly correlated with the energy of the incident ions in experiments involving laser-driven nuclear reactions. Using high-energy incident ions reduces the energy concentration of the generated neutrons. A novel “laser-collider” method was used at the Shenguang II laser facility to produce monoenergetic neutrons via ^7^Li (d, n) nuclear reactions. The specially designed K-shaped target significantly increased the numbers of incident d and Li ions at the keV level. Ultimately, 13.3 MeV neutrons were obtained. Considering the time resolution of the neutron detector, we demonstrated that the produced neutrons were monoenergetic. Interferometry and a Multi hydro-dynamics simulation confirmed the monoenergetic nature of these neutrons.

Fast neutrons, because of their unique characteristics of electrical neutrality and strong penetration capability in high-Z materials compared with charged particles and X-rays, possess tremendous appliciation value in the fields of materials testing for fusion power plant^1^, neutron radiography^2^, neutron therapy^3^, and others^4^,^5^. Intense neutron pulses can be obtained from spallation sources, nuclear reactors, and high-energy particle accelerators. Unfortunately, the enormous size and cost of these devices are preventing their widespread use. The rapid development of high-intensity laser technology offers an alternative approach at relatively low cost, and the ultra-fast nature of laser-driven neutron sources could allow this technology to find further applications in the ultrafast sciences. Because of their cross-sections and Q-values, three nuclear reactions are primarily used for neutron production:


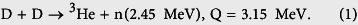



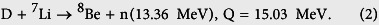



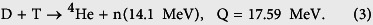


Because tritium is radioactive and not readily available, the (d, t) reaction, which is typically applied in inertial confinement fusion experiments, was excluded in the current study. Based on the remaining reactions, there are two main paths for ultra-fast laser-driven-neutron production. One possibility is to use the Coulomb explosion mechanism with a deuterium cluster target^6^,^7^. In this regime, the atoms of the clusters are entirely ionized by the laser-cluster interaction, which results in prompt explosion due to Coulomb repulsion and the production of the multi-keV ions required to initiate the fusion reactions. Extensive studies have indicated that 2.45 MeV quasi-monoenergetic neutrons can be produced using deuterium cluster targets through reaction (1)^8^,^9^,^10^,^11^,^12^. The measured energy spread, determined primarily based on the thermal velocity of the colliding ions, may be 10% or less^13^. However, when a cluster medium is used, the kinds of nuclear reactions available are restricted because it is impossible to transform all of the reaction materials (such as lithium or beryllium) into the cluster phase. Therefore, an alternative known as the “pitcher-catcher” approach has emerged^14^,^15^. In this method, the laser drives an ion beam (typically of deuterium ions) from a primary pitcher target, usually through target normal sheath acceleration (TNSA)^16^,^17^. Then, the ions collide with a secondary catcher target, where the nuclear reactions occur. In this way, high-energy neutrons can be obtained through the ^7^Li (d, n) and ^9^Be (d, n) nuclear reactions, as in (2)^18^,^19^. The laboratory energy E_3_ of a neutron produced in this way can be expressed as a function of the incident ion energy E_1_ and the angle θ of the emitted neutron relative to the incident ion,


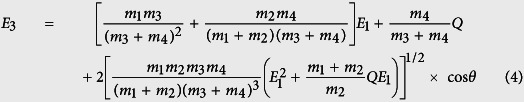


where m_1_, m_2_, m_3_, and m_4_ are the masses of the incident ion, the target particle, the neutron, and any other associated particles, respectively^20^,^21^. Equation (4) indicates that the energy E_3_ of neutrons produced in the pitcher-catcher regime strongly depends on the incident ion energy E_1_ and the Q-value of the nuclear reaction. Because the E_1_ values that can be achieved through the TNSA mechanism (usually tens of MeV) are on the same order as the Q-value, the neutron beams thus produced are not monoenergetic and therefore are not suitable for detector calibration, neutron-induced cross-section studies, or various other specialized applications^22^,^23^,^24^.

A novel method termed the “plasma-collider” technique was first applied by our group to enhance the neutron yields via the collision of high density deuterated plasma generated directly from the laser-plasma interaction^25^,^26^. This method has ability to produce numerous multi-keV ions participating in the nuclear reactions, although at a very low cross-section. Therefore, this method has enormous potential to be used to precisely calculate the cross-sections of ^7^Li (d, n) nuclear reactions because the incident ions are at the multi-keV level. It also has great potential to increase the probabilities of nuclear reactions induced in head-to-head collisions of plasma streams. In addition to these advantages, because E_1_ is several keV and this is negligible compared with the Q-value (on the order of MeV), equation (4) can be simplied as follows:


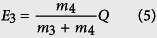


In this regime, the energy of the neutrons is determined simply by the Q-value of the nuclear reaction, and 13.3 MeV monoenergetic neutrons can be generated via ^7^Li (d, n) nuclear reactions. These 13.3 MeV neutrons can serve as a new type of laser-driven monoenergetic neutron source, in addition to 2.45 MeV and 14.1 MeV neutrons that can be produced through d (d, n) and d (t, n) nuclear reactions, respectively.

In this letter, we present new studies of the production of monoenergetic neutrons from laser-driven K-shaped D-Li target. The “plasma-collider” method was used to significantly increase the numbers of incident d and Li ions at the keV level. Experimental and theoretical methods were used to verify the production of 13.3 MeV monoenergetic neutrons via ^7^Li (d, n) nuclear reactions.

## Results

### Experimental setup

**Figure 1:**
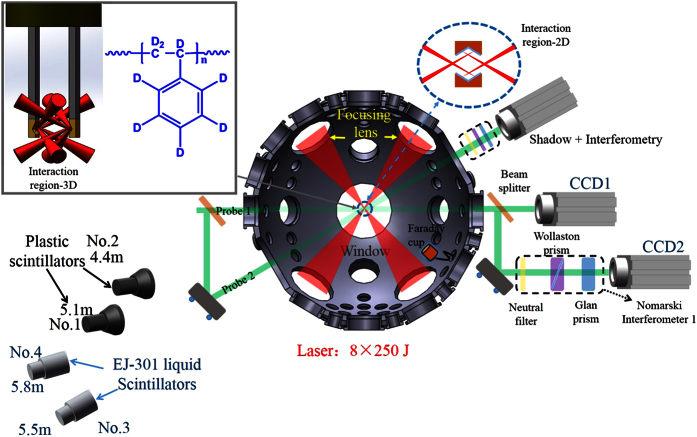
Experimental setup. Eight laser beams of laser were divided into two groups of four beams each, which were simultaneously focused onto the facing surfaces of the targets. Two types of K-shaped targets (D-Li and D-D) were employed. Two plastic and two EJ-301 liquid scintillator detectors were employed to measure the neutron yields via the TOF method. The process of plasma expansion and interaction was recorded by shadowgraph instruments and a Nomarski interferometer.

The experimental setup is shown in Fig. 1. Eight arms of main laser pulses delivered a total energy of nearly 2 kJ to the K-shaped D-Li or D-D target. The laser beams were divided into two groups of four laser beams each, which were simultaneously focused onto the facing surfaces of the target with a focal spot diameter of 150 μm. Another laser pulse with a wavelength of 526 nm and a 70 ps duration was used as a probe beam. By changing the delay time between the main laser pulses and the probe beam, the process of plasma expansion and interaction was recorded at different delay times using a Nomarski interferometer and shadowgraph instruments. Four neutron detectors were employed to measure the neutron spectrum via the time-of-flight (TOF) method. The detectors were numbered from 1 to 4 and located at 5.1 m, 4.4 m, 5.5 m, 5.8 m, respectively, from the fusion plasma. All of the TOF signals were recorded by oscilloscopes with a bandwidth of 1 GHz.

### Experimental results

**Figure 2:**
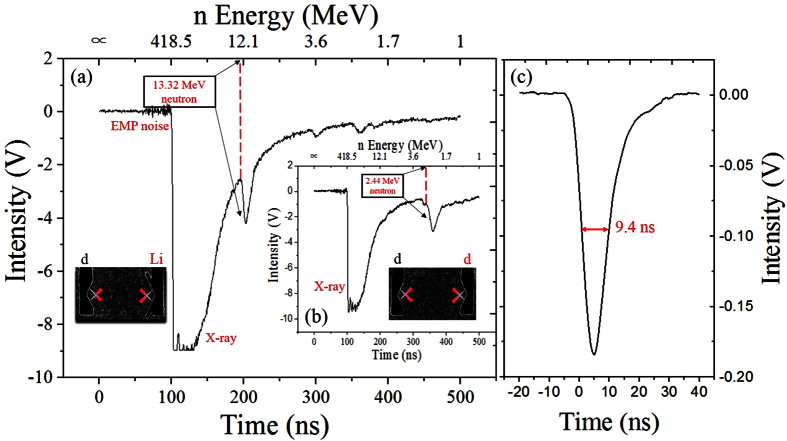
Experimental results. TOF results for 13.36 MeV and 2.45 MeV neutrons from liquid scintillator detector No. 4. (**a**) K-shaped D-Li target irradiated with eight laser beams. (**b**) K-shaped D-D target irradiated with eight laser beams. (**c**) Typical signal induced by a cosmic ray particle under natural conditions.

A high density plasma with numerous energetic deuterium ions and lithium ions was generated by the laser beams focused on the two-sided target. The plasma streams from both sides expanded toward the opposite target face and collided with each other. During this process, the deuterium ions completely blended together with the lithium ions and collision events occurred, resulting in great potential for the occurrence of ^7^Li (d, n) nuclear reactions. Figure 2 shows sample TOF signals obtained from detector No. 4. The neutron detection threshold was calculated to be 3 × 10^4^ by considering the detection length, active detection area, and detection efficiency of the detector. Figure 2(a) shows the typical neutron results from the D-Li target. The initial X-ray peak shows a falling edge at 98.8 ns. Considering the distance between detector No. 4 and the fusion plasma as well as the speed of X-rays in the atmosphere, the initial instant of laser-plasma interaction was accurately calculated to be 79.7 ns. The narrow neutron peak not far behind the large X-ray peak has a falling edge at 194.4 ns which corresponds to the energy of 13.32 MeV. There is a tiny deviation between the measured energy and the intrinsic energy (13.36 MeV), which may originate from the measurement error in the neutron flight distance. When the resulting ^8^Be nuclei were in their first or second excited states, a small amount of 10.7 MeV or 3.27 MeV neutrons could have been generated in the experiment. However, no such neutrons were detected in this shot because of the low yield. The pulse duration of the neutron peak in Fig.2(a) is 9.6 ns (full width half maximum, or FWHM). Figure 2(b) presents the TOF signal from D-D target as a reference. The falling edge that neutron peak starts at a time point which is corrosponding to the energy of 2.44 MeV. This is a direct proof that d (d, n) nuclear reactions happened. There is also a tiny energy deviation read from the intrinsic energy (2.45 MeV) which is coming from the measurement error as mentioned above. By calculating the area of the neutron signals and considering the same detector parameters used in ref. 25, we estimated the neutron yields from the d (d, n) nuclear reactions to be ~10^6^. Again considering the neutron signal from the ^7^Li (d, n) nuclear reactions, the calculated area of the D-Li neutron signal is nearly 6 times lower than in the D-D case. Thus, the neutron yields from the ^7^Li (d, n) nuclear reactions were greater than 10^5^. Figure 2(c) shows a typical cosmic ray signal recorded by liquid scintillator detector No. 4 under natural conditions. An individual cosmic ray particle was collected and left a sharp peak on the oscilloscope. The acquired pulse duration of the cosmic ray particle was 9.4 ns (FWHM), which indicates the inherent time resolution of the detector. In other words, the neutrons produced in the ^7^Li (d, n) nuclear reactions and the cosmic ray particle elicited detector responses of nearly the same time duration. This is strong evidence that the neutrons we obtained were quasi-monoenergetic.

**Figure 3:**
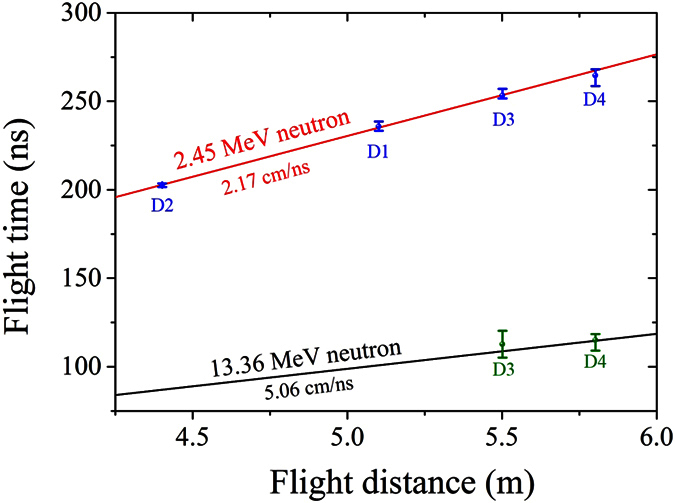
Experimental results. Flight time versus flight distance in relation to the 13.36 MeV and 2.45 MeV neutrons.

To further illustrate the neutron energy acquired in the experiments, the neutron signals from several laser shots and different detectors were analyzed. The black curve in Fig. 3 gives a plot of calculated flight time versus flight distance in relation to the 13.36 MeV monoenergetic neutrons corresponding to Fig. 2(a). Similarly, the red curve shows the case of 2.45 MeV neutrons. From the absolute zero time, the 13.36 MeV neutrons started from the plasma interaction region with speed of 5.06 cm/ns (2.17 cm/ns for 2.45 MeV neutrons). At a special time, the neutrons arrived at the detector positions. The circles in Fig. 3 represent the mean value of flight time acquired from the detectors in several laser shots, and the error bar gives the variation range of these TOF data. The results implied that the experimental flight time points were highly matched with the calculated flight time curves and the measured neutron energy was confirmed by the nearly consistent flight times of different neutron detectors. The No. 1 and No. 2 detectors were not available during the D-Li experiments.

**Figure 4:**
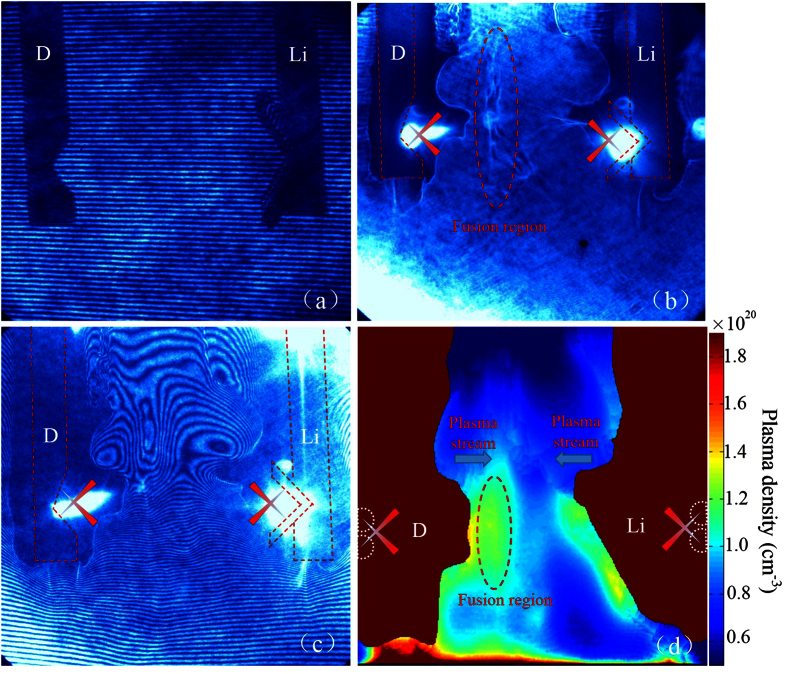
Optical imaging results. Optical imaging of the K-shaped D-Li target. (**a**) Interferogram of the original D-Li target. (**b**) Shadowgraph of the D-Li target at 3 ns after laser irradiation. (**c**) Interferogram of the D-Li target at 3 ns. (**d**) Spatial distribution of the plasma density at 3 ns, corresponding to (**c**).

The Nomarski interferometer and shadowgraph instruments were used to obtain a visual representation of the plasma expansion and interaction at a specific point in time after the laser-plasma interaction. Figure 4(a) shows the interferogram for the original D-Li target, which provides a reference for density analysis. The black area without fringes corresponds to the target body. The deuterium target is on the left side, and the LiF target is on the right side. Figure 4(b) shows a shadowgraph of the D-Li target at 3 ns after irradiation. The extension of the black area is caused by the expansion of the high-density target plasma. When the plasma density is higher than a critical density of ~4 × 10^21^ cm^−3^, the probe beam cannot penetrate the plasma. Therefore, the color depth represents the plasma density. There is a distinct opaque area at the center of Fig. 4(b), where the plasma density is higher than the density of the surrounding area. In this region, the plasmas were strongly fused and ^7^Li (d, n) nuclear reactions occurred. Figure 4(c) shows an interferogram of the D-Li target at 3 ns. The displacement of the interferometric fringes in Fig. 4(c) indicates density fluctuations in that area. The intense displacement of the fringes was analyzed via Abel inversion to determine the plasma density. Figure 4(d) shows the spatial distribution of the plasma density at 3 ns, corresponding to Fig. 4(c). The crimson area corresponds to the no-fringe area in Fig. 4(c), where the plasma density is higher than the critical density. This area was manually cropped, and was not considered in the Abel inversion. The figure shows two plasma streams with a plasma density higher than 10^20^ meeting at the center. In this region, the deuterium ions completely blended together with the lithium ions and collision events occurred, resulting in a great potential for the occurrence of ^7^Li (d, n) nuclear reactions.

### Simulation results

**Figure 5:**
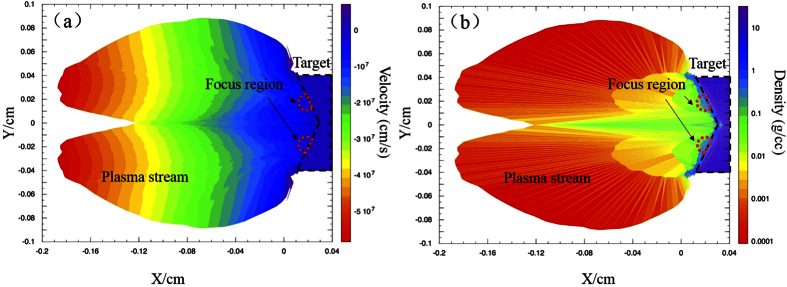
MULTI 2D simulation results. Spatial distributions of plasma velocity (**a**) and plasma mass density (**b**) at 3 ns after laser irradiation obtained from a MULTI 2D hydro-dynamics simulation.

The velocity and density results calculated from a simulation were used to verify the production of monoenergetic neutrons using the laser-collider method. Figure 5 shows the results of a MULTI 2D hydro-dynamics simulation of the K-shaped LiF target. The two-dimensional process of plasma expansion after laser irradiation was simulated. The K-shaped target appears on the right-hand side of the plots, as marked by a black dashed line. Two laser beams were focused on the K-shaped target, one on the upper part and one on the lower part. The plasma streams thus were produced and moved in the negative direction on the X axis. In Fig. 5, the X and Y axes represent the two spatial dimensions. The coordinates (X,Y) represent the position of the plasma. Figure 5(a) shows the spatial distribution of the plasma velocity at 3 ns. The color scale represents the velocity of the plasma stream. The highest velocity is 6 × 10^7^ cm/s, which corresponds to the lithium ion kinetic energy of 13 keV. According equation (5), the calculated energy spread is less than 0.1%. The head-to-head collision significantly enhanced the cross section for ^7^Li (d, n) nuclear reactions. This provided sufficient conditions for monoenergetic neutron production. Figure 5(b) shows the spatial distribution of the plasma mass density at 3 ns. The color scale in this figure represents the plasma density. High-density plasmas containing energetic lithium ions were produced in the simulation. The two figures together show that the K-shaped target generated an overdense region along the X axis in the middle of the figure because of the overlap of the two plasma streams from the upper and lower parts of the K-shaped target, which had an opening angle of 120°. With this mechanism, a K-shaped target is distinctly advantageous for promoting a high plasma density compared with other alternatives^25^. This enhancement of the plasma density significantly increases the collision strength of the plasma streams, thereby also providing increased opportunities for the collision of deuterium and lithium ions and giving rise to essential conditions for monoenergetic neutron production.

## Discussion

In conclusion, we have presented experimental results of the irradiation of a K-shaped D-Li target with 2 kJ laser pulses. Laser-driven 13.3 MeV monoenergetic neutrons were produced for the first time. The interferogram shows plasma expansion and a head-to-head collision state after laser irradiation, with a plasma density of up to 10^20^ cm^−3^ in the fusion region, guaranteeing a significant increase in the neutron yield. Theoretical simulation results strongly support the abundant production of monoenergetic neutrons. This study has demonstrated that the “laser-collider” method can be widely applied for monoenergetic neutron production. In the near future, this novel method will also be used to fill the gap of a ^7^Li (d, n) nuclear reaction cross section at the keV level, which is of great interest in the field of nuclear physics.

## Methods

### Laser system

The experiments were conducted at the Shenguang II laser facility in the National Laboratory on High Power Lasers and Physics. Eight arms of main laser pulses with energies ranging from 200 to 260 J and a 1 ns pulse duration delivered a total energy of nearly 2 kJ to the target^27^. The laser beams, with a wavelength of 351 nm (3ω), passed through the focusing lens on the chamber wall to form a focal spot of 150 μm in diameter (FWHM) at the target point, resulting in an average laser intensity of 10^15^ W/cm^2^. Another laser pulse with a wavelength of 526 nm and a 70 ps duration was used as a probe beam. Vacuum was established inside the experimental chamber was at ~10^−4^ Torr. The main laser pulses were separated into two groups, each composed of four laser beams; the two groups were focused to the surfaces on opposite sides of the target, as shown in the inset. This shooting pattern is depicted with only two laser beams on each side in Fig. 1 (Interaction region-2D), because the other two laser beams are concealed from the perspective of the image. The insert labeled “Interaction region-3D” in Fig. 1 provides clearer visual display. The probe beam was split into probe 1 and probe 2 while passing through the beam splitter prism, and probe 2 had a greater optical path difference than probe 1 by 2 ns. By changing the delay time between the main laser pulses and the probe beams, the process of plasma expansion and interaction was recorded at different delay times using the Nomarski interferometer and shadowgraph instruments.

### Target

Two types of 2-sided K-shaped targets with an opening angle of 120° and a separation distance of 2.5 mm were employed. One consisted of a 20 μm thick layer of deuterated hydrocarbon (CD_n_, the ratio of carbon to deuterium in the CD_n_ molecules was 1:1.3) and a 100 μm thick layer of lithium fluoride (LiF), and coated onto each facing surface of the K-shaped copper base (D-Li). The other was a 20 μm thick D-D target, which was used as a reference to gauge the TOF spectrum of the neutrons.

### Neutron radiation diagnostics

Four neutron detectors including two plastic and two EJ-301 liquid scintillator detectors were employed to measure the neutron spectrum using the time-of-flight (TOF) method. The operational parameters of these detectors were similar to those used in ref. 25. The two plastic scintillators were designed to have a larger neutron receiving area (π × 16 × 16 cm^2^) than the two liquid scintillators. The plastic detectors, No. 1 and No. 2, were located at 5.1 m and 4.4 m, respectively, from the fusion plasma. The two liquid scintillator detectors, with dimensions of π × 6.3 × 6.3 cm^2^, were more sensitive to neutrons because of the application of a high-voltage of up to 2 kV to the photomultiplier tubes (PMTs). The liquid detectors, No. 3 and No. 4, were located at 5.5 m and 5.8 m, respectively, from the fusion plasma. A Faraday cup was placed 44 cm away from the fusion region inside the chamber to collect energetic deuterium and lithium ions. All of the TOF signals were recorded by oscilloscopes with a bandwidth of 1 GHz.

### Hydrodynamics simulation

A two-dimensional hydrodynamics simulation was performed using the MULTI code^28^,^29^. The equation of states (EOS) data for the target materials (Li and Cu) were generated using MPQeos (a sub-program contained in the MULTI 2D code, developed by the Max Planck Institute for Quantum Optics) and the opacity data were obtained using the Steady-state Non-local thermal equilibrium OPacities (SNOP) code that is part of the MULTI code. The simulation geometry was a cone instead of the actual “>” shape because cylindrical symmetry is assumed in the 2D MULTI code. The simulation was performed only for the upper half of the cone and the remaining lower part was constructed through symmetry.

The beam profile was flat in time and Gaussian in space centered at 200 μm away the center of the K-shape. The simulation target was a concave copper cone coated with a Li layer of 100 um in thickness. The cone angle was 120° in total.

The horizontal resolution of the Li layer in the simulation was 5.8 μm (100 μm/20 cells/sin(π/3)), and the vertical resolution was 3.3 μm (400 μm/120 cells), whereas the resolution of the substrate Cu layer was 80 cells in the horizontal direction.

## Additional Information

**How to cite this article**: Zhao, J. R. *et al.* A novel laser-collider used to produce monoenergetic 13.3 MeV ^7^Li (d, n) neutrons. *Sci. Rep.* **6**, 27363; doi: 10.1038/srep27363 (2016).
